# Nasal splinting and mouth breathing training reduce emergence delirium after endoscopic sinus surgery: a randomized controlled trial

**DOI:** 10.1186/s12871-023-02262-2

**Published:** 2023-09-07

**Authors:** Hongjiao Xu, Zhenyuan Shen, Yuyu Gu, Yaodan Hu, Jihong Jiang, Xiang Li, Yanfang Zhao, Minmin Zhu, Jinbao Li

**Affiliations:** 1 Department of Anesthesiology, Shanghai General Hospital of Nanjing Medical University, 100 Haining Road, Hongkou District, Shanghai, 200080 China; 2 Department of Medical Affairs, Gumei Community Health Service Center, Shanghai Medical College Fudan University, Shanghai, China; 3 Department of Anesthesiology, Shanghai Jiaotong University First People’s Hospital (Shanghai General Hospital), Shanghai, China; 4 Department of Health Statistics, Second Military Medical University, Shanghai, China

**Keywords:** Emergence delirium, Functional endoscopic sinus surgery, General anesthesia, Randomized controlled trial

## Abstract

**Background:**

Emergence delirium (ED) is generally occurred after anesthesia associated with increased risks of long-term adverse outcomes. Therefore, this study aimed to evaluate the efficacy of preconditioning with nasal splint and mouth-breathing training on prevention of ED after general anesthesia.

**Methods:**

This randomized controlled trial enrolled 200 adult patients undergoing ESS. Patients were randomized to receive either nasal splinting and mouth breathing training (n = 100) or standard care (n = 100) before surgery. The primary outcome was the occurrence of ED within 30 min of extubation, assessed using the Riker Sedation-Agitation Scale. Logistic regression identified risk factors for ED.

**Results:**

Totally 200 patients were randomized and 182 aged from 18 to 82 years with 59.9% of males were included in the final analysis (90 in C-group and 92 in P-group). ED occurred in 16.3% of the intervention group vs. 35.6% of controls (P = 0.004). Male sex, smoking and function endoscopic sinus surgery (FESS) were independent risk factors for ED.

**Conclusions:**

Preoperative nasal splinting and mouth breathing training significantly reduced the incidence of emergence delirium in patients undergoing endoscopic sinus surgery.

**Trial Registration:**

ChiCTR1900024925 (https://www.chictr.org.cn/index.aspx) registered on 3/8/2019.

## Background

Emergence delirium (ED, aka emergence agitation, EA), which referring to a short-term state of dissociation of consciousness during the recovery from general anesthesia, manifested with motor agitation, incoherence, inconsolability, and unresponsiveness, is the most common neuropsychiatric complications after surgery [1–3]. The incidences of ED in patients underwent different surgeries varied from less than 10% up to 80% [4–6]. Although ED is often considered self-limited without the necessity of medical treatment, the agitated behavior sometimes may result in post-anesthesia care unit (PACU) accidents such as loss of catheters and tubes, accidental injuries, and elongated occupation [7, 8]. In addition, ED may be associated with increased risks of long-term adverse outcomes, including cognitive dysfunction and negative behavior that cause lower quality of life, readmission to hospitals, and even deaths [9–11]. Therefore, application of interventions in reducing the incidence of ED is still of great clinical significance.

Multiple factors related to characteristics of patients, anesthesia, and surgery have been linked with ED risks [12]. Generally, ED may be generated by an imbalance between the patient’s arousal state and the recovery of consciousness [13]. Therefore, factors related to patients’ cognitive functions and those cause agitation during induction and emergence of anesthesia may be associated with ED risks, for example, age, pre-operative anxiety, types of operation, postoperative pain, differential types and clearance methods of anesthetic agents [6, 14–16]. Accordingly, interventional strategies aiming at controlling various risk factors have been developed to reduce the incidence of ED, including medical interventions such as gabapentin [13], dexmedetomidine [17–19], melatonin [20] and ramelteon [21], as well as non-pharmacologic interventions such as time and place orientation, transcranial direct current stimulation or aided hearing and vision optimization [22].

Functional endoscopic sinus surgery (FESS) is a minimally invasive technique used to restore sinus ventilation and normal function [23]. Absorbable foams are regularly left in the nasal cavity after FESS until self-dissolvement to prevent postoperative adhesions and bleeding [24]. During the emergence from anesthesia, obstruction by these foams, in combination with postoperative mucosal edema in the nasal cavity, can cause breathing difficulties and feelings of suffocation which forced patients to breathe through mouth [25, 26]. These sensory abnormalities may cause an emergence agitation and increase the risk of ED. Previous studies have shown that preoperative adaption to postoperative abnormal sensory agitations may reduce the incidence of ED. Recent study showed that visual preconditioning for visual disturbance with an eye mask effectively reduced ED after eye surgery [27, 28]. Therefore, we designed a randomized controlled study to evaluate the efficacy of preconditioning by nasal splint and mouth-breathing training on preventing ED after FESS.

## Methods

### Study design

This is a randomized, single blind, parallel, controlled study to evaluate the efficacy of nasal splinting and mouth-breathing training in preventing ED in adults after FESS. Adult patients were 1:1 randomly allocated to receive preconditioning treatment or control treatment. The random sequences were generated by computer and concealed until the patients were included. Patients in the preconditioning treatment group received nasal splint and mouth-breathing training before FESS, and no any interventions were performed in the control group. The study protocol has been approved by the Shanghai General Hospital Institutional Review Board (IRB 2019KY039) and was registered prior to patient enrollment at chictr.org.cn (ChiCTR1900024925) [29].

### Patients

Patients scheduled for FESS at the Shanghai General Hospital, China from September 18th, 2019 to December 16th, 2020 due to chronic sinusitis were screened for eligibility. Inclusion criteria were: age over 18 years old; American Society of Anesthesiologists Classification (ASA) class I–II with no serious cardiovascular disease; and normal preoperative liver and kidney function. Patients were excluded if they met the following criteria: ASA III-IV, severe cardiovascular disease, or poor blood pressure control; history of mental illness, neurological diseases, or usage of sedative or antipsychotic drug; nasal malformation, history of nasal trauma or implantation of nasal prosthesis. Written informed consent was obtained from all participants.

### Interventions

**Fig. 1:**
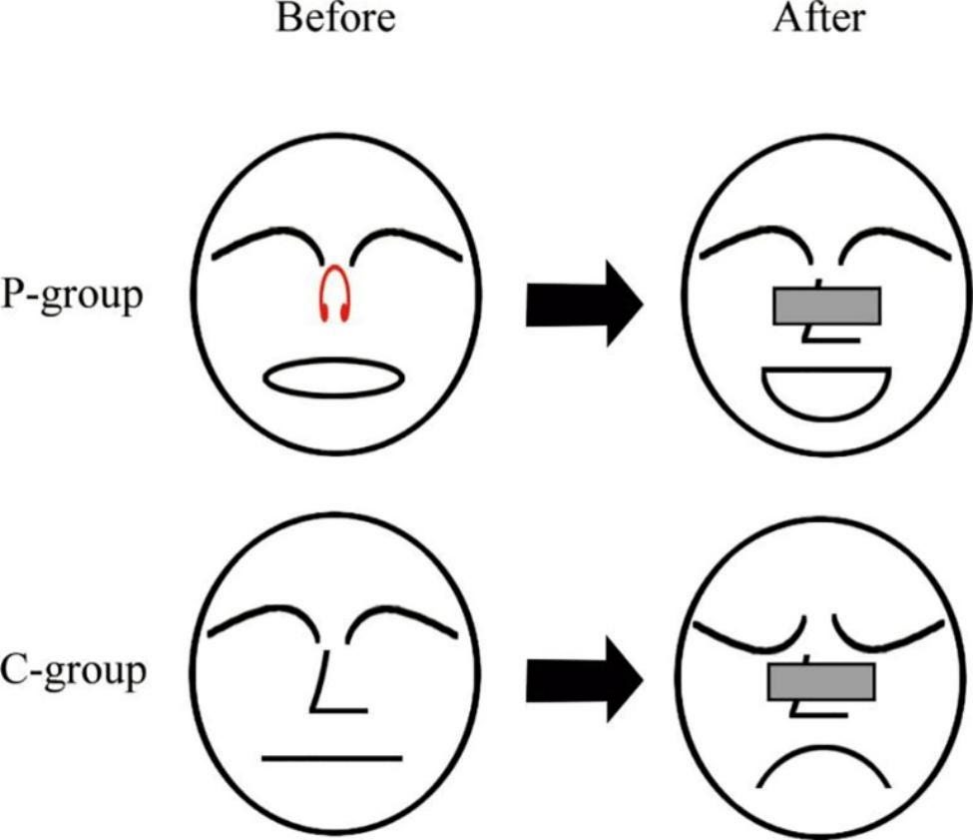
A graphic summary displaying that preconditioning by nasal splint and mouth-breathing training reduces emergence delirium after FESS

Patients in preconditioning group underwent nasal splint preconditioning and mouth breath training in pre-anesthesia room 1 h before surgery. In details, a nasal splint (patent No. 202020982582.9, China) was used to clamp the subnasal cartilage in front of the nasal cavity to block breathing through nose (Fig. 1). Patients were instructed to stay calm and adapt to breathing through mouth. Oxygen saturation (SpO_2_), heart rate (HR) and non-invasive blood pressure (NBP) were monitored during the process. If SpO_2_ level was below 95% of that at baseline and last for at least 5 min, the patient was considered to fail the adaption of mouth breathing training. For these patients, 4 L/min O_2_ was supplied until the SpO_2_ level returns to the normal level. Unless patients asked due to intolerance, the nasal splint was not removed until the anesthesia-induced loss of consciousness was detected. Patients in control group did not receive any interventions before the general anesthesia.

For general anesthesia induction, all patients administrated midazolam (0.05 mg/kg), propofol (2 mg/kg), and sufentanil (0.25 µg/kg). Rocuronium(0.6 mg/kg) was then administered. After satisfactory muscle relaxation, tracheal intubation was carried out, followed by 60% O_2_ ventilation and EtCO_2_ maintaining between 35 and 45 mmHg. Intraoperative anesthesia was maintained with desflurane inhalation, and the minimum alveolar concentration (MAC) value was controlled between 0.8 and 1.0. Before performing the nasal endoscopy, a dose of 5 or 10 µg sufentanil was administered depending on patient’s weight and circulation status. Due to the short duration of FESS, dose of sufentanil is usually not increased intraoperatively. In case the operation lasted for more than 1 h or the level of NBP and HR raised from inadequate anesthesia, another 5 or 10 µg sufentanil was further administered. If the operation wound was relatively large, 5 µg sufentanil was added at the end of the operation before nasal packing with absorbable foam. The package was composed of a 2 × 2 × 4 cm cuboid absorbable swelling sponge, which was inserted into the nasal cavity from the nostrils. Neostigmine antagonist was given according to the level of patient’s muscle relaxation. When tidal volume (Vt) reached to 5 ml/kg, the respiratory frequency (f) reached to 12/min, obvious swallowing reflex occurred, and the oxygen saturation maintained at 95% and above after 5 min of breathing, it was considered indication for extubation. In case sympathetic excitement caused by adrenaline infiltration in the nasal cavity, nimodipine and esmolol were used to control blood pressure and HR within the normal range.

### Outcomes

Outcomes assessed followed the statements of previously published protocol [29]. The primary outcome was the occurrence of ED, which was assessed within 30 min after extubation according to the Riker Sedation-Agitation scale [30] and a score of 5–7 points was considered as occurrence of ED.

The main secondary outcome was the severity of ED, which was determined based on the presences of combative behavior, thrashing, and hyperactive motor behavior [31]. If the above mentioned behavior occurred during stimulation, such as phlegm, but stopped at the removal of stimulation, the severity of ED was rated as mild. ED which occurred without stimulation that lasted for at least 5 min, but did not require any interventions, was considered as moderate. ED lasted for at least 5 min and had to be controlled with medication and/or physical restraint was rated as severe.

Other secondary outcomes included intraoperative monitored parameters, such as duration of surgery, duration and dosage of anesthesia, usage of nasal package; as well as those recorded in post anesthesia care unit (PACU), including time to extubation, physical and biochemical characteristics when leaving PACU and length of hospital stay. Pain was assessed using Numerical Rating Scale (NRS) every 5 min immediately after extubation by a trained anesthesia nurse. A reassessed score of 4 or above indicated substantial pain, which was managed by an intravenous injection of 3–5 µg sufentanil.

### Sample size

Based on the results from our pilot study, the incidence of ED after FESS was 21.3%. We assumed that the preconditioning would result in reduction of ED occurrence with an odds ratio (OR) of 1/3. At the setting of α = 0.05, an estimated sample size of 164 could provide a 90% of statistical power. Considering a potential dropout rate of 20%, 197 patients were required. Therefore, recruitment of 200 patients was planned.

### Randomization and blinding

Random number sequence was generated by computer. Single-blind ED assessment was performed by an independent anesthesiologist who was not aware of the grouping information (started observation until after the removal of nasal clip) and a nurse anesthetist (Yuyu Gu) at PACU, with necessary consultation with the intraoperative anesthesiologist.

### Statistical analyses

According to the intention-to-treat principle, we used full analysis data set to analyze the primary outcome. The full analysis set (FAS), including all patients underwent randomization and at least once training and with once measurement after surgery, was used for analysis of primary and secondary outcome. Normal distributed continuous data were presented as mean ± standard deviation (SD), and difference between groups was tested by using student t-test. Skew-distributed data was presented as median and the 25th percentile and 75th percentile, and between-group differences were tested by using Mann-Whitney test. Categorical variables were described as number (n) and percentage (%) and chi-square test or Fisher’s exact test was used for comparisons between the two groups. Multivariable logistic regression model was performed to identify independent associated factors of ED. All statistical analyses were conducted by using IBM SPSS Statistics (Version 20.0, IBM, Armonk, NY, USA). Two-sided P-value less than 0.05 indicated statistical significance.

## Results

**Fig. 2:**
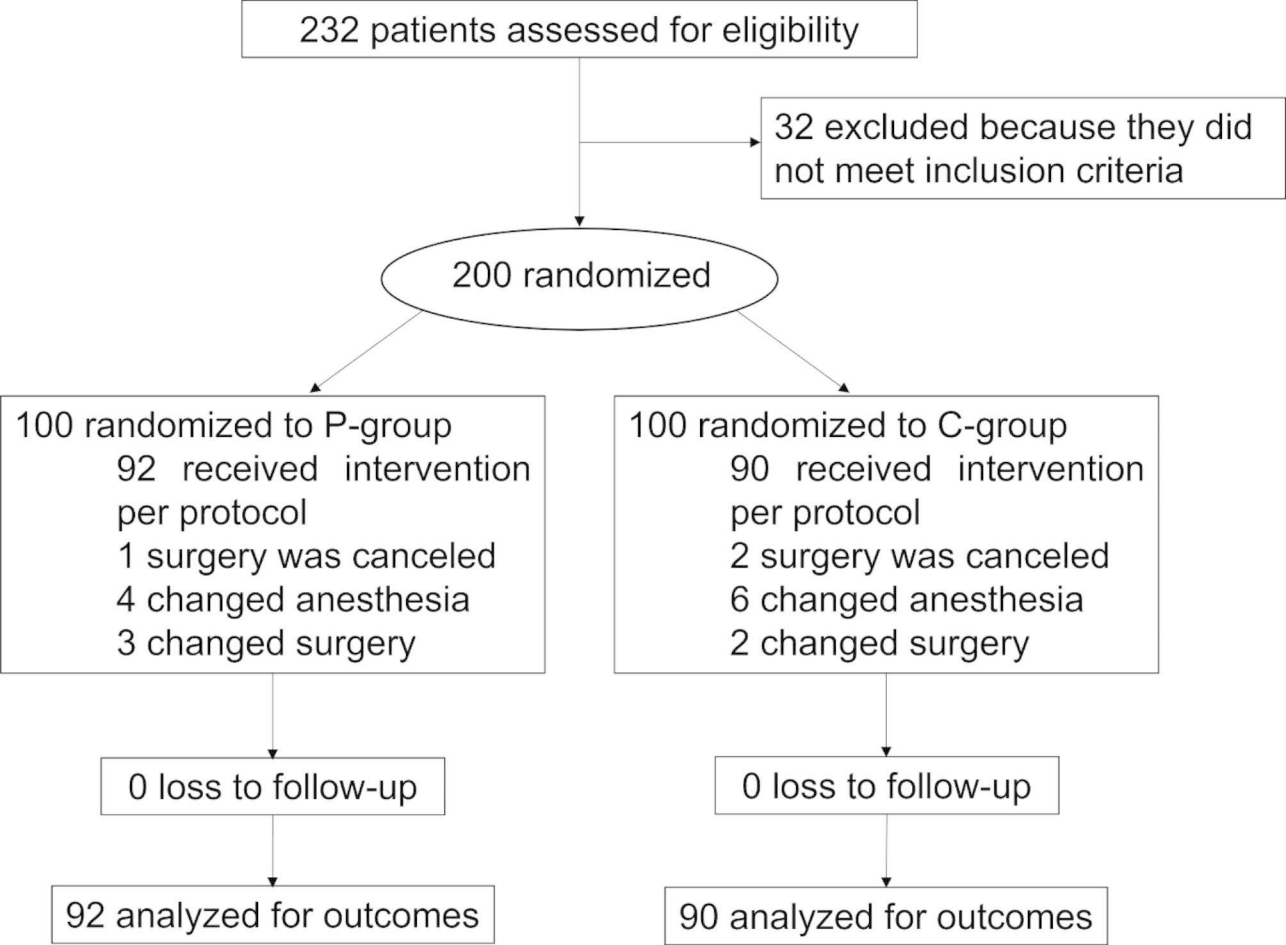
Recruitment, randomization, and patient flow diagram of the trial. P-group, group with preconditioning of nasal splint and mouth breathing training; C-group, control group

After screening of 232 patients, 200 met the inclusion and exclusion criteria and finally randomized. Among them, 18 patients were withdrawn (Fig. 2) due to cancellation of surgery (n = 3), changes of anesthesia (7 changed to local anesthesia, 2 changed to total intravenous anesthesia, and 1 performed no endotracheal intubation), or changes of surgery (2 with ear surgery, 1 with nasopharyngeal carcinoma radical operation, 1 with inferior turbinate and palatopharyngoplasty, and 1 with tonsil surgery). A total of 182 patients (90 in the control group and 92 in the preconditioning group) were included in the final FAS of analysis. Among patients in preconditioning, nasal splint was removed for 1 before anesthesia due to intolerance to mouth breathing training. In addition, extubation was failed for 1 patient in preconditioning group due to high risk of reflux aspiration, and 1 in the control group was observed with blindness as surgical complication. All these 3 patients were retained in PACU for more than 1 h.

The median age of 182 patients was 51.5 years and 59.8% were male (Table 1). The baseline demographic and clinical characteristics were comparable in two groups (all P-values > 0.05). In addition, no significant differences were observed in preoperative vital signs of HR, MAP and SpO_2_ between the two groups (All P-values > 0.05).

**Table 1:** Baseline characteristics of patients between preconditioning group and control group

Characteristics	Total (n = 182)	Control group (n = 90)	Preconditioning group (n = 92)	P-value
Demographic characteristics				
Age, year	51.1(18, 82)	53(18, 82)	51(20, 78)	0.547
Gender, N (%)				> 0.999
Male	109(59.9)	54(60.00)	55(59.78)	
Female	73(40.1)	36(40.00)	37(40.22)	
BMI, kg/m^2^	24.6 ± 3.95	24.7 ± 4.32	24.5 ± 3.57	0.767
Current smoking, N (%)	37(20.3)	15(16.7)	22(23.9)	0.270
History of HBP, N (%)	43(23.6)	20(22.2)	23(25.0)	0.728
History of DM, N (%)	14(7.7)	10(11.1)	4(4.35)	0.101
**Disease conditions**				
MAP at admission, mmHg	98.4 ± 11.8	97.4 ± 11.3	99.5 ± 12.2	0.221
ASA, N (%)				> 0.999
I	112(61.5)	55(61.1)	57(62.0)	
II	70(38.5)	35(38.9)	35(38.0)	
Head ache, N (%)				> 0.999
Negative	94(72.3)	45(72.6)	49(72.1)	
Positive	36(27.7)	17(27.4)	19(27.9)	
Nasal obstruction, N (%)				0.374
Negative	103(56.6)	54(60.0)	49(53.3)	
Positive	79(43.4)	36(40.0)	43(46.7)	
Nasal obstruction side, N (%)				0.786
Single	23(40.4)	11(44.0)	12(37.5)	
Both	34(59.6)	14(56.0)	20(62.5)	
Nasal obstruction description, N (%)				0.455
Intermittent	9(81.8)	5(100)	4(66.7)	
Persistent	2(18.2)	0(0)	2(33.3)	
Nasal obstruction with nasal polyps, N (%)				0.373
Negative	44(55.7)	18(50.0)	26(60.8)	
Positive	35(44.3)	18(50.0)	17(39.5)	
Nasal obstruction with septum deviation, N (%)				0.405
Negative	63(79.7)	27(75.0)	36(83.7)	
Positive	16(20.3)	9(25.0)	7(16.3)	
Chronic rhinosinusitis with nasal polyps, N (%)				0.749
Negative	126(69.2)	61(67.8)	65(70.7)	
Positive	56(30.8)	29(32.2)	27(29.5)	
**Preoperative vital signs**				
HR before Nasal splint, bpm	75.5 ± 11.2	74.4 ± 9.82	76.6 ± 12.4	0.184
MAP before Nasal splint, mmHg	95.1 ± 11.2	95.1 ± 10.4	95.0 ± 11.9	0.936
SpO_2_ before Nasal splint, %	99.0(98.0,99.0)	99.0 (98.0, 99.0)	98.5(98.0, 99.0)	0.052
h after Nasal splint, bpm	-	-	76.0(65.3, 81.0)	-
MAP after Nasal splint, mmHg	-	-	93.0(88.0, 98.0)	-
SpO_2_ after Nasal splint, %	-	-	99.0(98.0, 99.0)	-

Note: Continuous data were presented as mean ± SD or median (the 25th percentile, the 75th percentile). Categorical variables were displayed in number (percentage). BMI: body mass index; MAP: mean arterial pressure; ASA: American Society of Anesthesiologists; HBP: high blood pressure; DM: diabetes mellitus; HR: heart rate; SpO_2_: oxygen saturation. A P-value < 0.05 indicates significant difference between the two groups

The incidence of ED in the preconditioning group was 16.30% (n = 15) and significantly lower than control group (n = 32, 35.56%, P = 0.004) (Table 2). The severity of ED in precondition group appeared to be similar in two groups (control vs. preconditioning: 6.25% vs. 6.67 for severe and 21.9% vs. 13.3% for moderate degree, P = 0.449). The median intraoperative sufentanil dosage used in preconditioning group was 20 µg (P_25_, P_75_: 15, 20), which was significantly higher than 15 µg (P_25_, P_75_: 15, 20) in control group (P = 0.020). Other intraoperative measurements, including duration of surgery, duration of anesthesia, intraoperative sufentanil per unit anesthesia time, FESS with septoplasty, nasal package, and package side were similar in two groups (all P-values > 0.05). 2 patients in the control group (2.22%) and 1 patient in the preconditioning group (1.09%) had used the medication in PACU (P = 0.619). The time to extubation and the time of PACU were similar in two groups (all P-values > 0.05), the median time to extubation were 16.0 min (the 25th percentile, the 75th percentile: 12.0, 17.8) for preconditioning group and 17.0 min (12.0, 20.0) for control group respectively; and the time of PACU were 55.0 min (47.0, 59.0) and 56.0 min (47.0, 59.0), respectively. All other postoperative measurements, such as NRS in PACU, HR when left PACU, MAP when left PACU, SpO_2_ when left PACU, and postoperative hospital stays, were similar between two groups (All P-values > 0.05).

**Table 2:** Comparisons of outcomes between preconditioning group and control group

Outcomes	Preconditioning group(n = 92)	Control group (n = 90)	P-value
Major outcomes			
ED, N (%)			0.004
Negative	77(83.7)	58(64.4)	
Positive	15(16.3)	32(35.6)	
Degree of ED, N (%)			0.449
Mild	12(80.0)	23(71.9)	
Moderate	2(13.3)	7 (21.9)	
Severe	1(6.67)	2(6.25)	
**Intraoperative measurements**			
Duration of surgery, min	45.0(30.0, 68.8)	40.0(30.0, 60.0)	0.225
Duration of anesthesia, min	68.0(50.0, 88.0)	60.0(50.0, 79.3)	0.161
Intraoperative sufentanil dosage, µg	20.0(15.0, 20.0)	15(15.0, 20.0)	0.028
Dosage of sufentanil per unit anesthesia time, µg/min	0.27(0.27, 0.36)	0.27(0.22, 0.36)	0.562
FESS with septoplasty, N (%)			0.645
Without	82(89.1)	78(86.7)	
With	10(10.9)	12(13.3)	
Nasal package, N (%)			> 0.999
Negative	25(27.2)	24(26.7)	
Positive	67(72.8)	66(73.3)	
Package Side, N (%)			0.666
Single	13(25.0)	16(29.6)	
Both	39(75.0)	38(70.4)	
**Postoperative measurements**			
Medication in PACU, N/P			0.619
Negative	91(98.9)	88(97.8)	
Positive	1(1.09)	2(2.22)	
Time to extubation, min	16.0(12.0, 17.8)	17.0(12.0, 20.0)	0.714
PACU time, min	55.0(47.0,59.0)	56.0(47.0,59.0)	0.689
NRS in PACU	0(0, 6.00)	0(0, 6.00)	0.949
h when left PACU, bpm	71.0 (64.0, 77.5)	71.0(61.0, 78.0)	0.689
MAP when left PACU, mmHg	84.0(80.0, 91.5)	86.5 (79.0, 94.0)	0.815
SpO_2_ when left PACU, %	99.0(98.0, 99.0)	99.0(98.0, 99.0)	0.235
Postoperative hospital stays, day	3.00(3.00, 4.00)	4.00(3.00, 4.00)	0.308

Note: Data were presented as median (IQR) or number (percentage). ED: emergence delirium; PACU: post-anesthesia care unit; NRS: numerical rating scale; HR: heart rate; MAP: mean arterial pressure; SpO_2_: oxygen saturation. A P-value < 0.05 indicates significant difference between the two groups

A post-hoc sensitivity analysis identifying risk factors for ED was performed by using the univariate and multivariate models. After univariate models, we found totally 10 factors, including age, gender, BMI, smoking status, nasal obstruction with septum deviation, nasal splint, FESS with septoplasty, time to left PACU, medication use in PACU, and postoperative hospital stays, were significantly related with ED incidence (P < 0.05) (Table 3). Considering the risk factors being confirmed by previous evidence and clinical experience, we further added duration of surgery and anesthesia, intraoperative sufentanil dosage, minutes to extubation, NAS in PACU, HR, MAP, and SpO_2_ when left PACU into our multivariable model. Since there was no patient with ED having used the medication in PACU, we excluded this factor from the multivariable model (Table 4). After controlling all above mentioned factors, the patients with ED were more likely to be younger (OR: 0.977, 95% CI: 0.954, 1.000, P = 0.028), males (OR: 2.762, 95% CI: 6.757, 1.134, P = 0.001), current smokers (OR: 2.547, 95% CI: 1.065, 6.091, P = 0.019), FESS with septoplasty (OR: 2.770, 95% CI: 1.108, 6.925, P = 0.029), with longer postoperative hospital stays (OR: 1.672, 95% CI: 1.121, 12.49, P = 0.012), and with nasal septum deviation with obstruction (OR: 6.000, 95% CI: 1.793, 20.07, P = 0.004) compared with patients without ED. Nasal splint was a protective factor for ED, with the OR of 0.270 (95% CI: 0.123, 0.591, P = 0.001).

**Table 3:** Univariate analysis of potential associated factors of emergence delirium

Characteristics	With ED (N = 135)	Without ED (N = 47)	P-value
Baseline characteristics			
Age, year	54(18–82)	45(19–76)	0.028
Gender, N (%)			0.001
Male	71(52.6)	38(80.9)	
Female	64(47.4)	9(19.2)	
BMI, kg/m^2^	24.2(21.5, 26.5)	25.9(22.7, 27.7)	0.042
MAP at admission, mmHg	97.8 (12.1)	100 (10.6)	0.180
ASA, N (%)			> 0.999
I	83 (61.5)	29(61.7)	
II	52(38.5)	18(38.3)	
Head ache, N (%)			0.183
Negative	67(69.1)	27(81.8)	
Positive	30(30.9)	6(18.2)	
Nasal obstruction, N (%)			0.306
Negative	73(54.1)	30(63.8)	
Positive	62(45.9)	17(36.2)	
Nasal obstruction side, N (%)			0.750
Single	17(38.6)	6(46.2)	
Both	27(61.4)	7(53.8)	
Nasal obstruction description, N (%)			0.491
Intermittent	7(87.5)	2(66.7)	
Persistent	1(12.5)	1(33.3)	
Nasal obstruction with nasal polyps, N (%)			0.270
Negative	37(59.7)	7(41.2)	
Positive	25(40.3)	10(58.8)	
Nasal obstruction with septum deviation, N (%)			0.004
Negative	54(87.1)	9(52.9)	
Positive	8(12.9)	8(47.1)	
Chronic rhinosinusitis with nasal polyps, N (%)			0.354
Negative	96(71.1)	20(63.8)	
Positive	39(28.9)	17(36.2)	
Current smoker, N (%)			0.011
Negative	114(83.8)	31(66.0)	
Positive	21(16.2)	16(34.0)	
History of HBP, N (%)			0.686
Negative	104(77.0)	35(74.5)	
Positive	31(23.0)	12(25.5)	
History of DM, N (%)			0.525
Negative	123(91.1)	45(95.7)	
Positive	12(8.89)	2(4.26)	
Preoperative measurements			
Nasal splint, N/P			0.004
Negative	58(43.0)	32(68.1)	
Positive	77(57.0)	15(31.9)	
HR before Nasal splint, bpm	76.0(68.0, 82.0)	76.0(68.0, 81.0)	0.918
MAP before Nasal splint, mmHg	94.10(11.051)	94.9(11.6)	0.933
SpO_2_ before Nasal splint, %	99.0(98.0, 99.0)	99.0(98.0, 99.0)	0.512
h after Nasal splint, bpm	76.0(65.0, 81.0)	78.0 (69.5, 79.0)	0.903
MAP after Nasal splint, mmHg	93.3 (10.4)	91.7 (13.3)	0.625
SpO_2_ after Nasal splint, %	99.0(98.0, 99.0)	99.0(98.0, 99.0)	0.353
**Intraoperative measurements**			
Duration of surgery, min	45.0(30.0, 60.0)	45.0(25.0, 70.0)	0.970
Duration of anesthesia, min	64.0(50.0, 84.0)	65.0(49.0, 89.0)	0.931
Intraoperative sufentanil dosage, µg	20.0(15.0, 23.0)	20.0(15.0, 20.0)	0.567
Dosage of sufentanil per unit anesthesia time, µg/min	0.27(0.22, 0.33)	0.27 (0.22, 0.33)	0.660
FESS with septoplasty, N (%)			0.036
Without	123(91.1)	37(78.2)	
With	12(8.89)	10(21.3)	
Nasal package, N (%)			0.851
Negative	37(27.4)	12(25.5)	
Positive	98(72.6)	35(74.5)	
Package Side, N (%)			0.805
Single	21(26.6)	8(29.6)	
Both	58(73.4)	19(70.4)	
**Postoperative measurements**			
Minutes to extubation, min	17.0(12.0, 19.0)	16.0(12.0, 17.0)	0.816
Medication in PACU, N (%)			0.016
Negative	135(100)	44(93.6)	
Positive	0(0)	3(6.38)	
Time to left PACU, min	54.0(46.0,58.0)	58.0(49.0,60.0)	0.003
NRS in PACU, Sum	0(0, 6.00)	0(0, 6.00)	0.619
h when left PACU, bpm	70.0(63.0, 78.0)	72.0(64.0, 80.0)	0.098
MAP when left PACU, mmHg	84.0(79.0, 93.0)	86.0(81.0, 92.0)	0.654
SpO_2_ when left PACU, %	99.0(98.0, 99.0)	99.0(98.0, 99.0)	0.593
Postoperative hospital stays, day	3.00(3.00, 4.00)	4.00(3.00, 4.00)	< 0.001

Note: Continuous data were presented as mean ± SD or median (the 25th percentile, the 75th percentile). Categorical variables were displayed in number (percentage). ED: emergence delirium; BMI: body mass index; MAP: mean arterial pressure; ASA: American Society of Anesthesiologists; HBP: high blood pressure; DM: diabetes mellitus; HR: heart rate; SpO_2_: oxygen saturation; PACU: post-anesthesia care unit; NRS: numerical rating scale; FESS: function endoscopic sinus surgery. A P-value < 0.05 indicates significant difference between the two groups

**Table 4:** Multivariate logistic regression analysis of independent associated variables of emergence delirium

Characteristics	OR	95% CI	P-value
Preoperative			
Age, year	0.977	0.954, 1.000	0.053
Male	2.762	6.757, 1.134	0.026
BMI, kg/m^2^	1.081	0.985, 1.186	0.099
Current smoker	2.547	1.065, 6.091	0.036
Nasal splint	0.270	0.123, 0.591	0.001
FESS with septoplasty	2.770	1.108, 6.925	0.029
Nasal septum deviation with obstruction	6.000	1.793, 20.07	0.004
**Intraoperative & postoperative**			
Duration of surgery, min	0.987	0.939, 1.037	0.594
Duration of anesthesia, min	1.014	0.996, 1.064	0.581
Intraoperative sufentanil dosage	0.980	0.927, 1.036	0.474
min to extubation	1.014	0.966, 1.064	0.581
NRS in PACU	0.988	0.918, 1.064	0.753
PACU time, min	1.012	0.986, 1.039	0.367
Postoperative hospital stays, days	1.672	1.121, 12.49	0.012
h when left PACU, bpm	1.027	0.992, 1.063	0.134
MAP when left PACU, mmHg	0.986	0.948, 1.026	0.495
SpO_2_ when left PACU, %	0.799	0.618, 1.032	0.085

Note: OR, odds ratio; 95% CI, 95% confidence interval; BMI, body mass index

## Discussion

This randomized controlled trial showed that preconditioning with nasal splint and mouth-breathing training preparation significantly decreased the incidence of ED after FESS by more than 50%. The intraoperative sufentanil dosage of the preconditioning group was significantly improved by 20.0 µg. Post-hoc analysis suggested that patients with ED required more postoperative hospital stay, and younger, males, with smoking status, with FESS with septoplasty, with aasal septum deviation with obstruction and without nasal splint were more likely to present ED.

Otorhinolaryngology operations, or surgeries involving the ear, nose, and throat (ENT), were reported to be associated with increased risks of postoperative agitation and ED [6, 32]. Previous studies have shown that incidence of ED after surgery of ENT almost tripled as that in all other surgery types [6, 33]. Although the underlying pathological mechanism of ED remains opaque, the agitation caused by sensory abnormalities during anesthesia emergence, which were often encountered for patients undergo ENT surgeries, may play an important role [33, 34]. Preconditioning to these sensory abnormalities may help to reduce the agitation. For example, it was suggested that feeling of suffocation during emergence from anesthesia was responsible for ED after head and neck surgery [35], and prophylactic conditioning with eyepatch could reduce ED for children undergoing cataract surgery [27]. The patient’s hypopharyngeal airspace is constrained during FESS [36]. During emergence from anesthesia, the suffocating sense and compulsory mouth breathing may be an agitating factor for patients that increases ED risk. Thus, preoperative conditioning to nasal obstruction and training of mouth breathing may help the adaption to the postoperative conditions therefore decrease the chance of developing ED. The effect of nasal preconditioning on ED reduction was consistent with the results from a prior randomized controlled trial, where the incidence of ED was reduced from 60 to 30% in patients underwent nasal surgery by preoperative nasal closure for 30 min [37, 38].

The identified incidence of ED in this study was 25.8%, which was consistent with the previously evidence. For example, a retrospective study reviewing 792 adults who underwent general anesthesia for nasal surgery reported the overall incidence of emergence agitation at 22.2% [39]. Most moderate and severe ED cases were in the control group, with moderate for 7 of total 9 and severe for 2 of total 3. Therefore, the efficacy of preconditioning might decrease the severity of ED while this effect need to be confirmed in future.

This randomized trial potentially balanced many baseline characteristics and preoperative parameters between two groups; however, the occurrence of ED could be influenced by numerous unknown factors. For example, the intraoperative dosage of sufentanil was significantly higher in preconditioning group than that in the control group. To further eliminate the possibility that the observed difference in ED incidence was due to differences in other factors rather than the preconditioning treatment, we applied a post-hoc case-control analysis to confirmed the independent risk factors of ED. Our results identified age, gender, smoking, FESS with septoplasty and surgery category were independent risk factors of ED, which were consistent from previous evidences [6, 39].

There were several limitations in this study. Firstly, the study was a single center study with relatively limited samples size, hence our conclusion might not be stable and extension of our conclusion to other patients should be verified in further multi-center or multi-regional, well-designed, large-scaled randomized trials. Secondly, in this study the identification of ED mainly depended on the subjective assessment from physicians based on the postoperative evens and intraoperative signs. Also, the blindness of intervention for intraoperative anesthesiologist could not be achieved. All these might raise the uncontrolled bias for outcome assessment and introduced the misclassification bias. More objective evaluation of ED by anesthesiologist and nurse with blindness at PACU was necessary. Thirdly, although this is a drug-free intervention and had almost no side effects, this study did not include a positive parallel control to eliminate the non-drug modality with pharmacological treatment (e.g., dexmedetomidine) [28, 38]. Fourthly, we observed a higher sufentanil dose in the preconditioning group, which might attenuate the treatment effect, however, we concluded a significant effect of nasal splint conditioning and mouth-breathing training before anesthesia on reducing the occurrence of ED incidence. Finally, due to the limited number of participants, the subgroups analysis is less statistical powerful to be applying, thus we can not to further detect the specific target population for the treatment. A RCT found clonidine preconditioning effective in reducing ED in opium abusers, which suggested that our nasal splint conditioning and mouth-breathing training before anesthesia might have a specific target population, which warrant further well-designed large-scale study.

## Conclusions

Our single-center randomized controlled study compare the patients with nasal splint conditioning and mouth-breathing training before anesthesia with patients without intervention and found that this non-pharmacological intervention was an efficacy strategy for reducing the occurrence of ED by 50% among adults undergoing FESS. This non-pharmacological intervention is safe and cost-effective and can be easily implemented in practice, which could be a potential benefit strategy for patients prepared for surgery, although the conclusion on other surgery and population should be firstly verified before applying.

## Data Availability

The datasets used and/or analysed during the current study are available from the corresponding author on reasonable request.

## Abbreviations

ASA: American Society of Anesthesiologists Classification
ED: emergence delirium
f: frequency
FESS: Functional Endoscopic Sinus Surgery
HR: heart rate
IQR: interquartile range
MAC: minimum alveolar concentration
NBP: non-invasive blood pressure
NRS: Numerical Rating Scale
OR: odds ratio
PACU: post-anesthesia care unit
SD: standard deviation
SpO_2_: Oxygen saturation
Vt: tidal volume
